# Effects of Drying Methods on Taste Components and Flavor Characterization of *Cordyceps militaris*

**DOI:** 10.3390/foods11233933

**Published:** 2022-12-06

**Authors:** Man Zhang, Suhui Xing, Cuncun Fu, Fan Fang, Jun Liu, Juan Kan, Chunlu Qian, Qingqing Chai, Changhai Jin

**Affiliations:** 1 College of Food Science and Engineering, Yangzhou University, Yangzhou 225127, China; 2 State Key Laboratory of Biobased Material and Green Papermaking, School of Food Science and Engineering, Qilu University of Technology, Shandong Academy of Sciences, Jinan 250353, China

**Keywords:** *Cordyceps militaris*, drying methods, taste components, GC-MS, GC-IMS

## Abstract

The influences of four drying methods (hot air drying (HAD), vacuum freeze drying (VFD), vacuum drying (VD) and intermittent microwave combined with hot air drying (MW-HAD)) on the taste profile and flavor characteristic of *Cordyceps militaris* were investigated. MW-HAD samples had the highest levels of umami taste 5′-nucleotides, bitter taste amino acids, and equivalent umami concentration (EUC) value. The aroma fingerprints and differences of dried *Cordyceps militaris* were established by GC-MS with odor activity values (OAVs) and GC-IMS with principal component analysis (PCA). GC-MS data showed that the predominant volatiles of dried samples were aldehydes, alcohols, and ketones. VFD samples had the highest amount of total aroma compounds and C8 compounds. Moreover, 21 aroma-active components (OAVs ≥ 1) were the main contributors to the flavor of dried *Cordyceps militaris*. The OAVs of 1-octen-3-one and 3-octanone associated with mushroom-like odor in VFD were significantly higher than other samples. Furthermore, a significant difference in flavor compounds of four dried samples was also clearly demonstrated by GC-IMS analysis with PCA. GC-IMS analysis revealed that VFD samples had the most abundant flavor compounds. Overall, MW-HAD was an effective drying method to promote umami taste, and VFD could superiorly preserve volatiles and characteristic aroma compounds in dried *Cordyceps militaris*.

## 1. Introduction

*Cordyceps militaris*, a very promising functional and industrial fungus or mushroom, has been widely used as food and medicine in China [1]. *Cordyceps militaris* contains diverse bioactive compounds, including cordyceps polysaccharides, cordycepin, adenosine, ergosterol, etc. [2]. Researchers have reported that *Cordyceps militaris* has various physiological functions, such as antioxidant [3], anti-inflammatory, and anti-tumor effects [4]. The increased consumption of *Cordyceps militaris* is not only attributed to the nutritional value, but also its delicious taste and unique flavor [5]. Moreover, umami taste and aroma are critical elements influencing consumers acceptability, with the increase in people’s awareness about food organoleptic properties. Thus, it is necessary to reveal the comprehensive information on how processing affects the flavor properties of *Cordyceps militaris*.

Fresh *Cordyceps militaris* deteriorates readily and has a short shelf life, owing to its high moisture content (85.95%). Drying is an efficient processing method to preserve agricultural products and maintain food quality [6,7]. Drying methods most often used include hot air drying (HAD), vacuum drying (VD), vacuum freeze drying (VFD), and intermittent microwave-hot air drying (MW-HAD). HAD is the most adopted technique; it dehydrates foodstuffs by oven with a steady flow of hot air. It is easy to perform and low-cost, but it requires long drying time and has low energy utilization [8]. VD involves drying materials below the standard atmospheric pressure and evaporating the moisture under low temperature. It is often used in heat- and oxygen-sensitive products [9]. VFD is a well-accepted drying method. The samples are first frozen, and then the solid ice is removed by sublimation in a vacuum process [10]. This technique is considered superior because it can prevent the destruction of thermolabile components and maintain the nutrition, flavor, color, and texture of the product [11,12]. Li et al. [13] reported that VFD treatment could better preserve the taste compounds of *Pleurotus eryngii*. Microwave and hot air are usually applied in combination for drying products, which have the advantages of both MW and HAD [14]. The application of microwaves can shorten drying time and save energy consumption. Wang et al. [15] reported that MW-HAD-treated shiitake mushrooms had a predominant nitric aroma and moderate level of aldehydes and ketones associated with fresh-like characteristic when compared with HAD and infrared drying. Different drying methods have their own characteristic and may bring out the changes in flavor substances in mushrooms [6,16]. However, the effect of commonly used drying technologies on the formation and changes in flavor compounds in *Cordyceps militaris* has not been deeply studied.

Flavor induced by tasty and odorous chemicals is an important quality indicator of edible fungi [13]. Umami primarily caused by free amino acids (FAAs) and 5′-nucleotides contributes to the pleasant taste of mushrooms. The equivalent umami concentration (EUC) calculated based on these components can represent the level of umami in mushrooms [13]. Aroma is another essential feature, and the determination of aroma has gained more attention in recent years. Gas chromatography-mass spectrometry (GC-MS) is a commonly used technology for the detection and identification of compounds in food [17]. Comparative studies on the flavor analysis of *Cordyceps militaris* in different drying methods detected through GC-MS have been reported in recent years. Wu et al. [18] compared the main aroma difference of infrared freeze-dried (IRFD) and traditional freeze-dried (TFD) *Cordyceps militaris* by GC-MS. Results revealed that IRFD resulted in better retention of 3-octanone, 3-octanol, 1-octen-3-ol, and 1,3-octadiene than TFD. Wu et al. [19] found that short drying time and low drying temperature facilitated the retention of aroma compounds in infrared dried *Cordyceps militaris* through GC-MS. Wu et al. [20] also analyzed the influence of microwave-assisted pulse-spouted bed freeze drying (MPSFD), freeze drying (FD), and HAD on flavor compounds of *Cordyceps militaris* using GC-MS and indicated that FD and MPSFD could preserve aroma compounds better than HAD.

The newly developed gas chromatography-ion mobility spectrometry (GC-IMS) is a sensitive technology for evaluating flavor substances. This method has the advantages of GC (high separation) and IMS (rapid response) [21]. GC-IMS has been broadly applied in food classification, food adulteration detection, food freshness evaluation, safety control, and the characterization of off-flavor in food [22]. Hou et al. [23] successfully established the volatile fingerprints of shiitake mushrooms treated by HAD, VFD, VFD combined with HAD through GC-IMS. Chen et al. [24] compared the effect of drying rate and degree in HAD treatment on flavor characteristic of shiitake mushrooms using GC-IMS and indicated that GC-IMS was an effective tool to discriminate different samples. Moreover, GC-IMS was also used to evaluate the volatiles of *Cordyceps militaris* chicken soup. Results demonstrated that the addition of *Cordyceps militaris* resulted in the disappearance of some aroma components, and the enzymolysis process led to the increase in some volatile substances [25]. As mentioned above, GC-IMS was a useful tool in the flavor evaluation of some edible mushrooms; however, the application of GC-IMS in *Cordyceps militaris* treated by different drying methods has never been reported.

Although several drying methods have been used in *Cordyceps militaris*, the specific information on how to obtain high-value and flavor-rich dried *Cordyceps militaris* products is lacking. Therefore, the objectives of this study were to analyze the influences of four drying methods (HAD, VFD, VD, and MW-HAD) on the flavor quality of *Cordyceps militaris*. The taste profile was evaluated by FAAs, 5′-nucleotides, and EUC value. The flavor fingerprints and discrimination analysis of four dried *Cordyceps militaris* samples were performed by GC-MS combined with OAVs and GC-IMS with PCA. The results obtained could provide reference for the preservation of fresh *Cordyceps militaris*, the further flavor analysis of dried *Cordyceps militaris*, and development of *Cordyceps militaris* processing products.

## 2. Materials and Methods

### 2.1. Materials and Chemicals

Fresh *Cordyceps militaris* (initial moisture content of 85.95%) with uniform size was purchased from Hongyu Agricultural Science Co. Ltd. (Xuzhou, Jiangsu, China). Then all samples were immediately kept in a refrigerator (4 °C). Before drying, the *Cordyceps militaris* were rinsed and drained. 1,2-Dichlorobenzene and n-alkanes (C_7_–C_40_) of chromatography grade were obtained from Sigma Aldrich Ltd. (St. Louis, MO, USA).

### 2.2. Drying Procedures

#### 2.2.1. Hot Air Drying (HAD)

HAD was conducted in a hot-wind circulation oven (GZX-GF 101-3 BS, Yuejin Medical Co., Shanghai, China). Fresh *Cordyceps militaris* samples (100 g) were dried at 50 °C with an air velocity of 1.0 m/s. The moisture content of *Cordyceps militaris* reached below 9.0% (wet basis). The total drying time was about 13 h.

#### 2.2.2. Vacuum Freeze Drying (VFD)

Fresh *Cordyceps militaris* (100 g) was initially frozen at −70 °C for 24 h in the freezer. Then, the samples were transferred to lyophilize in a laboratory-scale freeze dryer (LPHA1-2/LO-2, Bomaixing Co., Beijing, China) at −55 °C for 48 h [23]. The chamber pressure was 20 Pa. The moisture content of *Cordyceps militaris* reduced to 9.0% (wet basis).

#### 2.2.3. Vacuum Drying (VD)

For the VD process, fresh *Cordyceps militaris* (100 g) was dried using a vacuum drying oven (DZF-6500, Yiheng Co., Shanghai, China) equipped with a vacuum pump. The drying conditions were set at 50 °C under a vacuum degree of 0.85 MPa. Drying lasted for about 17 h, until the samples moisture reached below 9.0% (wet basis).

#### 2.2.4. Combined Microwave-Hot Air Drying (MW-HAD)

Fresh *Cordyceps militaris* (100 g) was firstly dried in a microwave oven (XMJD65 W-2, Jiequan Microwave Co., Nanjing, China) with a power output of 375 W until the moisture content reached 66.7% (wet basis). The drying time in the microwave was 40 min. They were then further processed by hot air drying until the sample’s moisture fell below 9.0% (wet basis).

### 2.3. Free Amino Acids Assay

FAAs composition and concentration of dried samples were analyzed by an Agilent 1100 liquid chromatograph according to Lan et al. [26]. Sample powders (1.0 g) with 25 mL of 5% trichloroacetic acid (TCA) were extracted with ultrasonic waves for 20 min. The solution was left at room temperature (about 20 °C) for 2 h then centrifuged (10,000× *g*, 30 min) and filtered. The collected liquid was used for further detection.

### 2.4. 5′-Nucleotides Assay

5′-Nucleotides of dried samples were detected following the method of Pei et al. [27] with some modifications; 0.5 g of sample powder was homogenized with distilled water and then boiled for 1 min. After the mixture centrifugation (4500× *g*, 15 min), the obtained supernatant was filtered through a 0.22 μm filter membrane and then used for analysis. The 5′-nucleotides were determined using a Waters 1525 EF HPLC system fitted with the Sunfire C18 column (4.6 mm × 250 mm, 5 µm) and a UV detector. The eluents were methanol/distilled water/H_3_PO_4_ (5/95/0.05, *v*/*v*/*v*) at a flow rate of 0.8 mL/min, and the UV detector wavelength was at 254 nm.

### 2.5. Equivalent Umami Concentration (EUC)

EUC represented the equivalent concentration of monosodium glutamate (MSG) provided by a mixture of umami amino acids and 5′-nucleotides. The value was calculated by the following equation [28]:

(1) $$\mathrm{EUC}=\Sigma a_{i}b_{i}+1218(\Sigma a_{i}b_{i})(\Sigma a_{j}b_{j})$$

where the EUC value was expressed in terms of g MSG/100 g; $a_{i}$ was the content (g/100 g) of Asp or Glu; $a_{j}$ was the content (g/100 g) of 5′-AMP, 5′-GMP or 5′-IMP; $b_{i}$ was the relative umami concentration (RUC) of Asp (0.077), Glu (1) related to MSG; $b_{j}$ was the RUC of 5′-AMP (0.18), 5′-GMP (2.3), and 5′-IMP (1). The synergistic constant based on the content was 1218 (g/100 g).

### 2.6. GC-MS Analysis

The volatile compounds of dried *Cordyceps militaris* were extracted by head space solid phase microextraction (HS-SPME) and detected using a GC-MS instrument (Trace ISQ, Thermofisher, Waltham, MA, USA) with a DB-5 MS column (30 m × 0.25 mm × 0.25 µm) following the method of Wu et al. [20] with a slight modification.

The sample (0.5 g) with 1,2-dichlorobenzene solution (internal standard, 10 μL, 5 mg/500 mL) was added into a 20 mL glass vial. The fiber (DVB/CAR/PDMS) was used to extract the volatiles from the vial in a thermostatic water bath at 55 °C for 30 min. Then, the fiber was inserted into the GC injection port and desorbed at 250 °C for 7 min. The oven temperature program employed was set at 40 °C initially and kept for 3 min, raised to 70 °C at 3 °C/min, then increased to 180 °C at 4 °C/min and maintained for 2 min, then finally 15 °C/min to 240 °C. The flow rate of helium was 1 mL/min. The mass spectra were obtained in an electron ionization at 70 eV and scanned in a range of 35–350 amu. The ionization source temperature was set at 200 °C. Volatiles were identified on the base of a comparison of mass spectra with the spectral data from NIST 11 and linear retention indexes (LRI) with values from databases or the literature. The LRIs of volatiles were calculated by the n-alkane standard under the same conditions. Volatile concentrations were calculated from the GC peak area of the samples related to that of the internal standard [29].

### 2.7. Odor Activity Value (OAV)

The OVA was the ratio of the measured concentration of flavor substances in the samples to its odor threshold value in water obtained from the literature [30]. The substance with OAV ≥ 1 had a contribution to the dried *Cordyceps militaris* aroma [31].

### 2.8. GC-IMS Analysis

The volatile components of dried *Cordyceps militaris* were determined by a GC-IMS instrument (FlavourSpec^®^, Dortmund, Germany) equipped with an autosampler, following Hou et al. [23]; 0.5 g of finely ground dried power with 5 mL of purified water was put into a 20 mL headspace bottle. The mixture was treated with ultrasound for 30 min for dissolution. Subsequently, the sample was incubated at 55 °C for 30 min. After that, 0.5 mL of headspace was injected into the injector through a heated syringe (85 °C). Then, an MXT-5 column (15 m × 0.53 mm × 1 μm) at 60 °C (column temperature) was used to separate the volatiles, a N_2_ (99.99%) carrier gas was used. The flow rate was as follows: 2 mL/min for 2 min, 10 mL/min for 8 min, 100 mL/min for 10 min, 150 mL/min for 10 min.

### 2.9. Statistical Analysis

Data are shown as means ± standard deviation (SD) by triplicate experiments. Analysis of variance (significance level *p* < 0.05) and the correlation analysis were carried out by SPSS 23.0 (SPSS Inc., Chicago, IL, USA). GC-IMS data were performed using VOCal software, three plug-ins, and library search software which could analyze samples from different angles. The three plug-ins included the reporter plug-in, gallery plot plug-in, and dynamic principal component analysis (PCA) plug-in. The first one was directly applied for the comparison of spectral differences between samples. The second one was used to compare the differences of flavor fingerprint. The third one was used for cluster analysis.

## 3. Results and Discussion

### 3.1. Free Amino Acids of Cordyceps militaris under Different Drying Methods

As shown in Table 1, 17 free amino acids (FAAs) were identified in the four dried *Cordyceps militaris*. The total content of FAAs in dried *Cordyceps militaris* ranged from 36.11 mg/g to 41.55 mg/g. The changes in amino acid content in the mushrooms were mainly caused by the release of amino acids, protein degradation, Strecker degradation of FAAs, or the Maillard reaction relating to FAAs and reducing sugars [27]. The content of total FAAs was the highest in HAD samples and the lowest in VFD samples, as freezing at the low temperature might prevent protein degradation to a certain extent, and dehydration might cause a loss of FAAs [32]. Similar results had been reported by Li et al. [13], in which the total number of FAAs in *Pleurotus eryngii* from the freezing drying process was lower than that from HAD and the vacuum drying process.

Amino acids were important and critical taste components in edible mushrooms. FAAs were divided into four categories on the basis of their taste characteristics: MSG-like or umami, sweet, bitter, and tasteless amino acids [13]. The MSG-like or umami taste, including Glu and Asp, was the most typical mushroom taste [33,34]. The content of MSG-like amino acids was relatively higher in HAD samples (8.82 mg/g) than other samples. The lowest concentration of MSG-like amino acids was detected in VD samples (5.10 mg/g). Results indicated that HAD treatment was better at preserving MSG-like amino acids than other drying methods. It was reported that MSG-like and sweet components mainly contributed to the natural taste of mushroom [35]. Ala, Gly, Ser, and Thr were sweet amino acids [16]. The contents of sweet amino acids in the four samples ranged from 13.84 mg/g to 16.84 mg/g, and the highest level was detected in HAD samples. From Table 1, Ala was the most abundant sweet amino acid in dried *Cordyceps militaris*, accounting for 60.68% to 64.42%. The MW-HAD samples had the highest level of bitter amino acids and the lowest level of sweet amino acids. However, the bitter attribute from bitter substances might be masked by the sweetness produced by sweet components [35].

### 3.2. 5′-Nucleotides of Cordyceps militaris under Different Drying Methods

As shown in Table 2, five types of 5′-nucleotides (5′-CMP, 5′-AMP, 5′-UMP, 5′-GMP, 5′-IMP) were detected in dried *Cordyceps militaris*. Total 5′-nucleotides contents in samples from four drying methods ranged from 7.99 mg/g to 8.33 mg/g, while the highest was found in MW-HAD samples. Among all five 5′-nucleotides, it could be observed that 5′-IMP was slightly effected by different drying methods. Moreover, 5′-UMP levels were the highest and accounted for 58.10% to 72.15% of total 5′-nucleotides.

5′-nucleotides were reported to contribute to MSG-like taste [5]. 5′-AMP provided the sweet taste and was a bitterness inhibitor with the ability to sweeten and diminish sour, astringent, bitter taste [36]. 5′-GMP had a meaty note and could enhance the flavor with its umami taste stronger than MSG [37]. Moreover, 5′-IMP was another taste-active compound in mushrooms, and it could enhance the flavor with other nucleotides [27]. As for MSG-like 5′-nucleotides, their contents were in the range of 1.25–2.19 mg/g in the four dried *Cordyceps militaris* samples. The highest content of MSG-like 5′-nucleotides was detected in MW-HAD samples. The flavor 5′-nucleotides (5′-GMP + 5′-IMP) levels ranged from 0.53 mg/g to 1.03 mg/g, while the highest level was found in MW-HAD treatment.

### 3.3. EUC of Cordyceps militaris under Different Drying Methods

**Figure 1:**
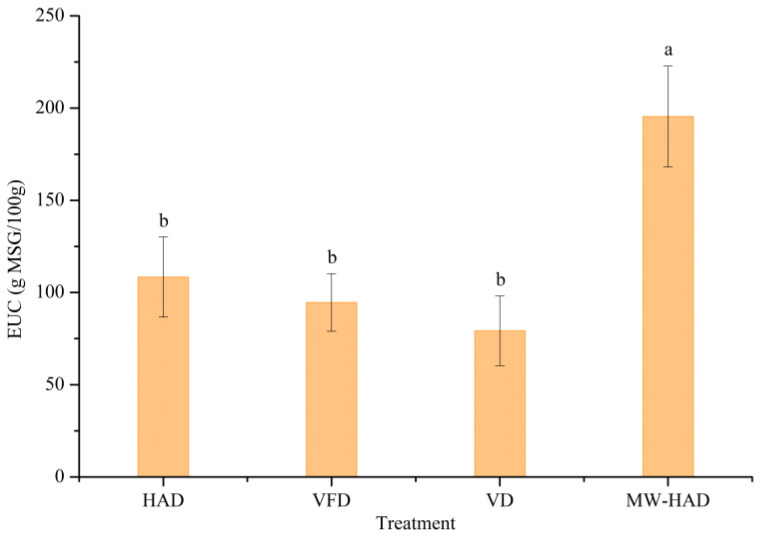
EUC value of *Cordyceps militaris* in different drying methods. Different lowercase (a, b) indicates significantly different (*p* < 0.05).

The umami taste of edible mushrooms could be enhanced by the cooperative effect of MSG-like amino acids and 5′-nucleotides [5,38]. The EUC value was used to evaluate the synergistic effect on umami character. From Figure 1, the EUC values of dried *Cordyceps militaris* ranged from 79.30 to 195.43 g MSG/100 g. EUC values could be divided into four levels on the basis of MSG/100 g: <10 g, 10–100 g, 100–1000 g, >1000 g [39]. EUC values of VFD and VD samples were at the second level. HAD and MW-HAD treatment increased the EUC values to the third level. As stated previously, *Cordyceps militaris* dried by HAD had the most MSG-like amino acids. MW-HAD generated the highest content of 5′-AMP and 5′-GMP. Among the four samples, *Cordyceps militaris* treated by MW-HAD had the highest EUC value of 195.43 g MSG/100 g, which indicated that MW-HAD was a potential drying technology for enhancing the umami intensity of dried *Cordyceps militaris*.

### 3.4. The Volatile Compounds of Cordyceps militaris under Different Drying Methods by GC-MS

**Figure 2:**
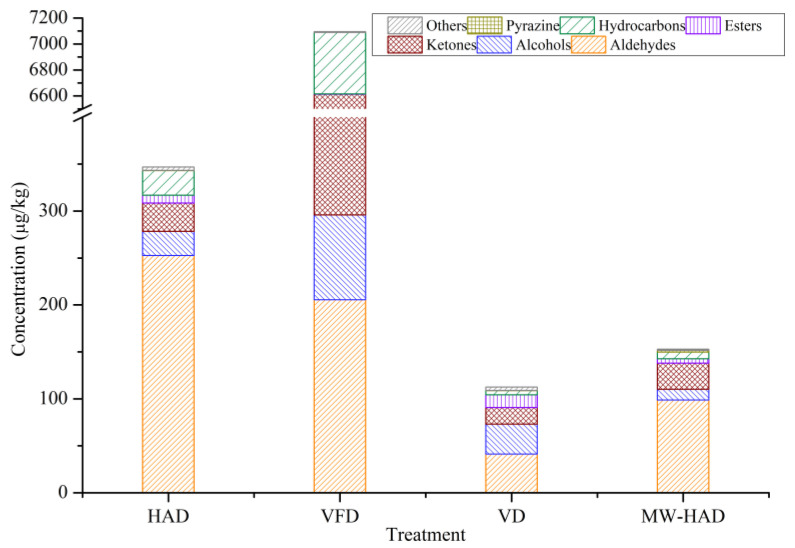
Concentrations of different chemical types of volatiles in *Cordyceps militaris* from four drying methods obtained by GC-MS.

As shown in Table 3 and Figure 2, 93 flavor substances were identified in dried *Cordyceps militaris*, including 19 aldehydes, 12 alcohols, 20 ketones, 14 esters, 20 hydrocarbons, 3 pyrazines, and 5 others. The total concentration of all flavor substances was different in samples processed by different drying methods. For VFD process, 38 flavor substances were identified, and the amounts of total volatiles were significantly higher than those in other dried samples. We could conclude that VFD treatment contributed to the retention of flavor substances in *Cordyceps militaris*. In our study, 1-octen-3-one, 3-octanone, and 1,3-octadiene were the most plentiful components in VFD samples which accounted for 93.95% of the total concentration of volatiles. Our result was similar to the previous study that 1-octen-3-ol, 3-octanone, 1,3-octadiene, and 3-octanol were detected as the most abundant components in freeze-dried *Cordyceps militaris* [20]. There were 51, 61, and 60 kinds of flavor substances detected in HAD, VD, and MW-HAD samples, respectively. VD-treated samples showed a lower concentration of total volatiles compared with those of HAD samples at a constant temperature, indicating that the vacuum process promoted the degradation or evaporation of flavor substances.

The predominant flavor substances in *Cordyceps militaris* processed by the four drying methods were aldehydes, ketones, and alcohols. The concentrations of aldehydes, ketones, and alcohols in HAD, VFD, VD, and MW-HAD were 308.85 μg/kg, 6612.86 μg/kg, 90.70 μg/kg, and 137.99 μg/kg, respectively. Aldehydes contributed to the overall aroma because they possessed a low odor threshold [40]. The highest content of total aldehydes was detected in HAD samples (252.65 μg/kg). The reason might be that aldehydes were primarily formed from the degradation and oxidation of unsaturated fatty acids, and a certain drying temperature promoted the production of aldehydes [39]. The more abundant aldehydes of dried *Cordyceps militaris* contained pentanal, benzeneacetaldehyde, (E)-2-octenal, nonanal, and hexanal. Pentanal, which had an almond, fruity, and sweet odor [41], was detected in the four samples, and its highest content was found in HAD samples. Benzeneacetaldehyde, which was described as having a floral and honey-like odor, was mainly produced by Strecker degradation of phenylalanine [42,43]. The highest content of benzeneacetaldehyde was detected in HAD samples and then followed by VFD samples. (E)-2-Octenal, which had a fruity note, was produced by the oxidation of fatty acids [44]. The concentration of (E)-2-octenal in VFD was significantly higher than those of other drying samples. The amounts of hexanal with a grass-like odor and nonanal with a fatty and soapy odor were higher in MW-HAD samples than other products [43]. Methional, which had a boiled-potato-like odor, was previously reported as one of the aroma-active components in pine-mushrooms [43]. Methional was only detected in HAD and VFD samples, and the highest concentration was found in HAD process.

The typical flavor characteristic of edible mushrooms was mainly induced by a series of C8 compounds, such as 3-octanone, 1-octen-3-one, 3-octanol, (E)-2-octen-1-ol, 1-octanol, phenylethyl alcohol, (E)-2-octenal, and so on, which were mainly derived from the oxidation of linoleic or linolenic acid [39,45]. 3-Octanone, which had a mushroom-like, earthy, and fruity odor [16], was only detected in VFD and VD samples, and VFD samples had the highest amount (5716.93 μg/kg). 1-Octen-3-one, described as a mushroom-like odor [43], which was the typical compound of mushrooms, was formed by the enzymatic degradation of linoleic and linolenic acids [46]. VFD samples had the highest amount of 1-octen-3-one (563.39 μg/kg), followed by HAD samples (13.94 μg/kg). 3-Octanol, which had a mushroom-like aroma [43], was only detected in VFD. (E)-2-Octen-1-ol with a mushroom-like odor reached the highest content in MW-HAD samples [43]. 1-Octanol had a chemical, sweet odor, and its highest content was detected in VFD. Phenylethyl alcohol, which had a floral and sweet odor [47], was detected in HAD samples (0.95 μg/kg), VD samples (0.18 μg/kg), and MW-HAD samples (0.21 μg/kg). The total amount of C8 compounds in VFD was significantly higher than those in HAD, VD, and MW-HAD. This was consistent with the literature that heat treatment might cause destructions of ketones and alcohols through non-enzymatic and enzymatic reactions during the drying process [48].

Esters were produced by the esterification of alcohols and acids. The short-chain and long-chain esters were reported to have a fruity odor and a slight fatty note, respectively [25]. VD samples had the highest level of esters. Hydrocarbons could result from decarboxylation and cleavage of carbon–carbon bonds in fatty acids [49]. 1,3-Octadiene was the most abundant compound and only detected at a high level in VFD samples (385.57 μg/kg). Pyrazines, derived from the Maillard reaction during the drying process, were only detected in HAD, VD, and MW-HAD samples [50]. MW-HAD samples had the most varieties and the highest content of pyrazines, indicating that the microwave coupled with hot air treatment facilitated the generation of pyrazines. Wang et al. [15] reported that intermittent MW-HAD treatment attained a higher content of pyrazines compared to HAD in dried shitake mushrooms. Although many types of esters, hydrocarbons, pyrazines, and other components were identified, they might be regarded as unimportant contributors to the odor of dried *Cordyceps militaris* samples due to their relatively low levels and high odor threshold values [20,25].

### 3.5. OAVs of Aroma-Active Compounds of Cordyceps militaris under Different Drying Methods

OAV represented the contribution of each flavor compound to the overall aroma profile. The compound with OAV more than 1 was considered to be responsible for the aroma [31,47]. The OAVs of odorants obtained by GC-MS in dried *Cordyceps militaris* samples are listed in Table 4. Twenty-one aroma-active compounds with OAVs ≥ 1 were detected in samples. These compounds contained fourteen aldehydes, three alcohols, three ketones, and one ester. Results revealed 15, 11, 14, and 16 aroma-active compounds in HAD, VFD, VD, and MW-HAD samples, respectively. For HAD samples, 1-octen-3-one had the highest OAV (871), followed by phenylethyl alcohol (48), 2,4-nonadienal (38), pentanal (34), (E,E)-2,4-decadienal (26), nonanal (26), methional (24), benzeneacetaldehyde (16), and 2-nonenal (16). In VFD samples, 1-octen-3-one had the highest OAV (35212), followed by 3-octanone (267), pentanal (28), methional (13), (E)-2-octenal (13), and benzeneacetaldehyde (11). For VD samples, 2-nonenal had the highest OAV (15), followed by 2,4-nonadienal (12). In MW-HAD samples, 1-octen-3-one had the highest OAV (295), followed by nonanal (38), 2-nonenal (26), and phenylethyl alcohol (11). It could be observed that 1-octen-3-one and 3-octanone, associated with mushroom-like odor, were the crucial aroma-active substances in dried *Cordyceps militaris*, especially for the samples processed by VFD.

In sum, different drying methods had a significant influence on the flavor substances and aroma-active compounds of *Cordyceps militaris*. VFD samples had strong mushroom odor. The changes of flavor characteristic in dried *Cordyceps militaris* might be associated with drying conditions, including low temperature for VFD, vacuum evaporation for VD, and relatively higher temperature for HAD and MW-HAD.

### 3.6. The Volatile Fingerprints of Cordyceps militaris under Different Drying Methods by GC-IMS

**Figure 3:**
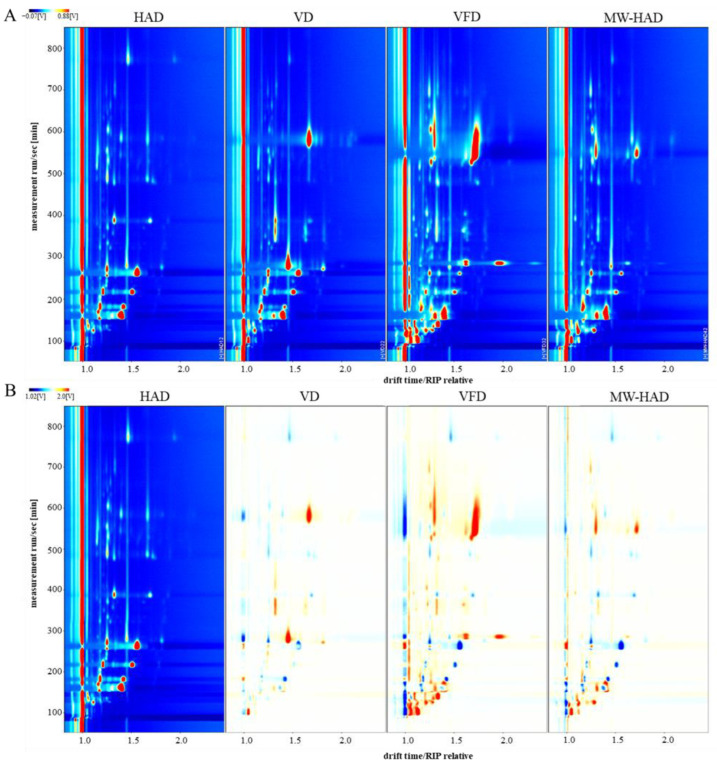
Topographic plot of *Cordyceps militaris* from four different drying methods ((**A**): Two-dimensional spectrogram of volatiles; (**B**): Differential spectrogram of flavor substances).

In our study, GC-IMS was applied to explore the flavor compounds in the four dried *Cordyceps militaris* samples. The overhead view of flavor substances in different dried samples is shown in Figure 3A. The ordinate and the abscissa represent the retention time of GC and ion migration time, respectively [51]. Moreover, each point on the fingerprint represents a volatile compound. The signal intensity of each compound mainly depends on the color; red indicates high concentration and white indicates low concentration. The darker the color, the greater the intensity and content of the compound. The variances of flavor substances in different samples were mainly induced by the signal number, position, ion signal intensity, and time of ion peaks [52]. From Figure 3A, the color point distribution of volatiles in four groups of samples were different, and most of the signals appeared in the retention time of 100–800 s and drift time of 1.0–2.0.

The different comparison model was carried out to differentiate the four dried samples. The HAD sample was taken as the control, and all the other samples were deducted from the control (Figure 3B). If the flavor components were consistent, the background was white, while a red color represented that the intensity of the compound was higher than that in the control, and blue color represented lower. As shown in Figure 3B, the concentration of VD volatiles was lower than that of the HAD sample, while the concentration of VFD and MW-HAD volatiles was lower than that of the HAD and also somewhat higher than the HAD sample within the drift time of about 1.0. When the drift time was in the range of 1.0–1.5, the concentration of most of VFD volatiles was significantly higher than that of the HAD sample, while the concentration of most of VD volatiles was lower than that of the HAD sample. In addition, most of the signals in the four dried *Cordyceps militaris* samples were located at the retention time of 100–300 s, and some of them appeared at the retention time of 500–700 s. It was reported that non-polar substances retained longer on non-polar columns than polar substances [42]. The concentration of some non-polar compounds in the VFD process was higher than that observed in other treatments. In all the four drying methods, the concentration of most of the VFD volatile compounds was higher than that of other drying methods. The improved intensity of some signals indicated that VFD treatment could better preserve the flavor compounds, which was consistent with the GC-MS results.

**Figure 4:**
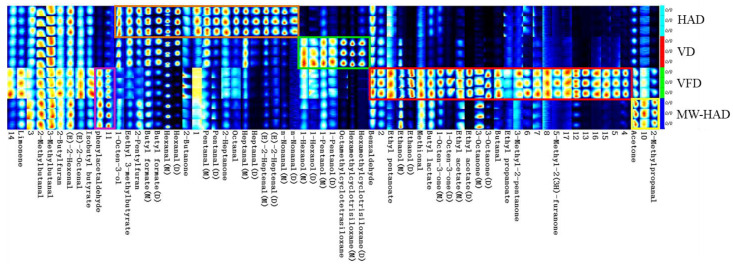
Fingerprint spectra of flavor compounds in *Cordyceps militaris* dehydrated by four different drying methods.

To further clarify the specific flavor substances in the four dried samples, the fingerprint plot of GC-IMS with all the selected peaks was shown in Figure 4. A total of 67 signal peaks were tentatively detected, and 38 volatile compounds were identified (fifteen aldehydes, four alcohols, seven ketones, seven esters, two furans, one terpene, and two others). The unidentified components were expressed by numbers, and some volatiles were identified with monomer (M) and dimer (D) in the fingerprint plot. This was also observed in GC-IMS fingerprints of shiitake mushrooms [23,24].

In the fingerprint plot, each row represents the signal peaks (volatile compounds) detected in one sample, and each column exhibits the difference of flavor component in different samples. As shown in Figure 4, limonene, 2-methylbutanal, 3-methylbutanal, 2-butylfuran, (E)-2-hexenal, (E)-2-octenal, and isobutyl butyrate were detected in all the four dried samples. Among these substances, the concentration of limonene, (E)-2-octenal, and isobutyl butyrate in VFD samples was relatively higher than those in other samples. Phenylacetaldehyde and one unidentified compound (11) with higher signal intensity were detected in both VFD and MW-HAD samples and marked as the purple region of their characteristic aroma compounds. 1-Octen-3-ol, ethyl 3-methylbutyrate, 2-pentylfuran, butyl formate M, butyl formate D, hexanal M, hexanal D, 2-butanone, pentanal M, pentanal D, 2-heptanone, octanal, heptanal M, heptanal D, (E)-2-heptenal M, (E)-2-heptenal D, nonanal M, and nonanal D were detected in HAD samples and labelled as the orange region of the samples’ characteristic compounds. A similar result was found in the report of Hou et al. [23], which showed that HAD treatment could increase the content of aldehydes (such as pentanal, hexanal, heptanal, (E)-2-heptenal) in dried shiitake mushrooms compared with VFD. 1-Hexanol M, 1-hexanol D, 1-pentanol M, and 1-pentanol D were detected in VD samples and labelled as the green area, which were the characteristic volatiles in the fingerprint plot. From Figure 4, benzaldehyde, ethyl pentanoate, ethanol M, ethanol D, methional, butyl lactate, 1-octen-3-one M, 1-octen-3-one D, ethyl acetate M, ethyl acetate D, 3-octanone M, 3-octanone D, butanal, ethyl propanoate, 3-methyl-2-pentanone, and 5-methyl-2(3H)-furanone were found in VFD samples and labelled as the red area of the samples’ characteristic flavor compounds. Acetone and 2-methylpropanal were detected in MW-HAD samples and marked as the yellow region of this samples’ characteristic substances. Overall, the fingerprint spectra demonstrated that the flavor substances in *Cordyceps militaris* from different drying methods were obviously different, and VFD samples had a higher abundance of flavor compounds than other samples.

### 3.7. Correlation and PCA Analysis

The correlation analysis between dried *Cordyceps militaris* samples using the flavor data obtained from GC-IMS is listed in Table 5. All the triplicate samples in the four drying groups had a high value of Pearson’s correlation coefficient, indicating that *Cordyceps militaris* in the same drying treatment had good repeatability. VFD exhibited a low correlation coefficient with HAD and VD, thus the distinctions between VFD and HAD, as well as VFD and VD were the largest. The correlation coefficient between VD and HAD, as well as MW-HAD and VFD was relatively higher, thus the differences were smaller.

**Figure 5:**
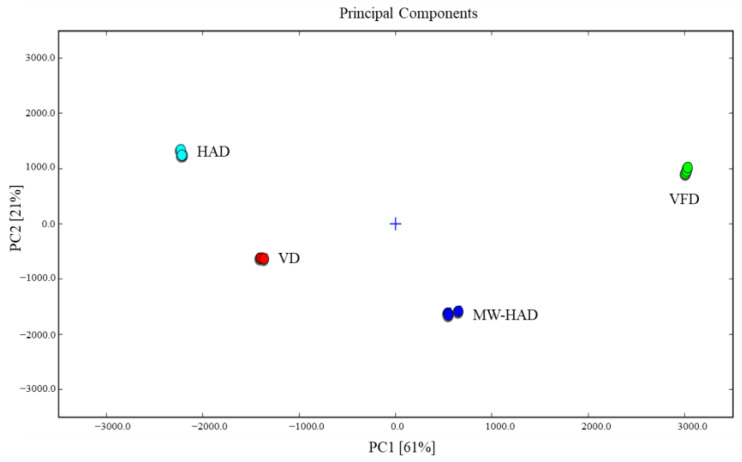
Dynamic PCA plot of *Cordyceps militaris* for four drying methods obtained from GC-IMS.

The multivariate analysis of PCA was applied to measure the differences among the four dried *Cordyceps militaris* samples based on the flavor compounds obtained from GC-IMS (Figure 5). The first two principal components explained 82% of the variation (PC1 explained 61%, and PC2 was 21%), which could reflect the most information relating to the samples. In general, when the distances between samples were far apart, the difference in volatiles was great, and if they were close, the difference was small. According to the distribution of samples, it could be clearly found that HAD, VD, VFD, and MW-HAD samples were well distinguished into four groups in the visualization, and the flavor differences among samples in different drying methods were obvious. VFD could be separated by the positive score values of PC1 and PC2. MW-HAD samples were located in the bottom right area. HAD samples were distributed in the upper left area. VD samples were located in the bottom left area. The results indicated that GC-IMS data could effectively distinguish *Cordyceps militaris* samples with different drying treatments.

## 4. Conclusions

This study conducted comparative analyses on taste components and flavor characteristics in *Cordyceps militaris* dried by HAD, VFD, VD, and MW-HAD methods. The dried *Cordyceps militaris* in the MW-HAD treatment had the highest levels of umami taste 5′-nucleotides, bitter taste amino acids, and EUC value. GC-MS results revealed that 93 kinds of volatiles were detected, of which 21 were aroma-active compounds with OAVs ≥ 1. The main flavor substances in dried *Cordyceps militaris* were aldehydes, alcohols, and ketones. Compared to HAD, VD, and MW-HAD, VFD treatment resulted in the highest amount of total aroma compounds and C8 compounds associated with fresh mushroom-like characteristics, which was better for the retention of flavor compounds. GC-IMS results demonstrated that 67 signal peaks were detected, and 38 volatile compounds were identified. VFD samples had higher signal intensities of aroma compounds in the flavor fingerprint of GC-IMS. The difference of the characteristic flavor substances in the four dried samples was obvious. The dynamic PCA analysis from GC-IMS data indicated that *Cordyceps militaris* in different drying methods could be clearly distinguished from each other. This study revealed that different drying methods played a crucial role in the flavor quality of dried *Cordyceps militaris*. The results obtained provide information on the flavor formation of dried *Cordyceps militaris* and the flavor enhancement to *Cordyceps militaris* products.

## Figures and Tables

**Table 1:** Content of free amino acids in *Cordyceps militaris* from different drying methods.

Free Amino Acids ^1^	Content (mg/g)			
	HAD	VFD	VD	MW-HAD
Asp	2.03 ± 0.02 ^a^	1.59 ± 0.05 ^b^	1.39 ± 0.09 ^c^	1.69 ± 0.05 ^b^
Glu	6.78 ± 0.03 ^a^	5.28 ± 0.06 ^c^	3.71 ± 0.08 ^d^	6.22 ± 0.04 ^b^
Ser	1.09 ± 0.01 ^b^	1.07 ± 0.01 ^b^	1.10 ± 0.02 ^b^	1.13 ± 0.01 ^a^
His	0.90 ± 0.02 ^c^	1.11 ± 0.01 ^b^	0.73 ± 0.04 ^d^	1.60 ± 0.02 ^a^
Gly	0.83 ± 0.02 ^b^	0.82 ± 0.04 ^b^	1.05 ± 0.04 ^a^	0.84 ± 0.04 ^b^
Pro	1.64 ± 0.02 ^d^	2.20 ± 0.00 ^c^	2.44 ± 0.01 ^a^	2.35 ± 0.01 ^b^
Arg	2.78 ± 0.02 ^a^	2.52 ± 0.04 ^c^	2.82 ± 0.02 ^a^	2.67 ± 0.02 ^b^
Ala	10.85 ± 0.02 ^a^	8.55 ± 0.03 ^d^	10.49 ± 0.01 ^b^	8.64 ± 0.03 ^c^
Tyr	2.07 ± 0.02 ^a^	1.79 ± 0.01 ^b^	1.60 ± 0.03 ^c^	2.06 ± 0.04 ^a^
Cys-s	0.02 ± 0.01 ^a^	0.01 ± 0.00 ^a^	0.00 ± 0.00 ^a^	0.01 ± 0.00 ^a^
Thr	4.08 ± 0.02 ^a^	3.66 ± 0.04 ^b^	3.65 ± 0.04 ^b^	3.22 ± 0.02 ^c^
Val	3.14 ± 0.02 ^c^	2.88 ± 0.02 ^d^	3.60 ± 0.04 ^a^	3.32 ± 0.02 ^b^
Met	0.28 ± 0.03 ^a^	0.19 ± 0.03 ^b^	0.12 ± 0.02 ^b^	0.14 ± 0.04 ^b^
Ile	0.42 ± 0.04 ^c^	0.56 ± 0.03 ^b^	0.66 ± 0.04 ^a^	0.70 ± 0.02 ^a^
Phe	1.17 ± 0.04 ^a^	0.79 ± 0.02 ^c^	0.99 ± 0.04 ^b^	0.94 ± 0.03 ^b^
Leu	1.31 ± 0.03 ^b^	1.21 ± 0.03 ^c^	1.43 ± 0.03 ^a^	1.45 ± 0.03 ^a^
Lys	2.16 ± 0.03 ^a^	1.88 ± 0.02 ^c^	1.98 ± 0.04 ^b^	1.80 ± 0.04 ^d^
MSG-like	8.82 ± 0.01 ^a^	6.87 ± 0.11 ^c^	5.10 ± 0.17 ^d^	7.92 ± 0.08 ^b^
Sweet	16.84 ± 0.08 ^a^	14.09 ± 0.12 ^c^	16.29 ± 0.11 ^b^	13.84 ± 0.10 ^d^
Bitter	10.01 ± 0.15 ^b^	9.27 ± 0.20 ^c^	10.34 ± 0.18 ^b^	10.81 ± 0.14 ^a^
Tasteless	4.24 ± 0.05 ^a^	3.67 ± 0.03 ^c^	3.58 ± 0.07 ^c^	3.86 ± 0.08 ^b^
Total	41.55	36.11	37.75	38.78

^1^ MSG-like (monosodium-glutamate-like), Asp + Glu. Sweet, Ala + Ser + Thr + Gly. Bitter, Arg + Met + Ile + His + Phe + Leu + Val. Tasteless, Lys + Tyr. Results were given as means ± SD, *n* = 3. Different superscripts (a–d) in the same row were significantly different (*p* < 0.05). HAD, hot air drying; VFD, vacuum freeze drying; VD, vacuum drying; MW-HAD, microwave with hot air drying.

**Table 2:** Content of 5′-nucleotides in *Cordyceps militaris* from different drying methods.

5′-Nucleotides ^1^	Content (mg/g)			
	HAD	VFD	VD	MW-HAD
5′-CMP	1.15 ± 0.11 ^ab^	1.06 ± 0.07 ^b^	1.55 ± 0.29 ^a^	1.31 ± 0.15 ^ab^
5′-AMP	1.06 ± 0.07 ^a^	0.62 ± 0.11 ^b^	0.68 ± 0.19 ^b^	1.15 ± 0.12 ^a^
5′-UMP	5.26 ± 0.19 ^b^	5.96 ± 0.05 ^a^	5.04 ± 0.20 ^bc^	4.84 ± 0.13 ^c^
5′-GMP	0.42 ± 0.10 ^b^	0.53 ± 0.11 ^b^	0.65 ± 0.14 ^b^	0.98 ± 0.18 ^a^
5′-IMP	0.11 ± 0.00 ^a^	0.09 ± 0.04 ^a^	0.07 ± 0.01 ^a^	0.05 ± 0.02 ^a^
MSG-like 5′-nucleotides	1.59 ± 0.18 ^b^	1.25 ± 0.18 ^b^	1.40 ± 0.35 ^b^	2.19 ± 0.04 ^a^
Flavor 5′-nucleotides	0.53 ± 0.11 ^b^	0.62 ± 0.06 ^b^	0.72 ± 0.15 ^b^	1.03 ± 0.16 ^a^
Total	8.00	8.26	7.99	8.33

^1^ 5′-CMP, 5′-cytosine monophosphate; 5′-AMP, 5′-adenosine monophosphate; 5′-UMP, 5′-uridine monophosphate; 5′-GMP, 5′-guanosine monophosphate; 5′-IMP, 5′-inosine monophosphate. MSG-like 5′-nucleotides: 5′-AMP + 5′-IMP + 5′-GMP; Flavor 5′-nucleotides: 5′-GMP + 5′-IMP. Different letters (a–c) in the same row represented significantly different (*p* < 0.05).

**Table 3:** Volatile compounds of *Cordyceps militaris* for different drying methods detected by GC-MS.

NO.	Compound	LRI ^1^	LRI ^2^	ID ^3^	Concentrations (µg/kg)			
					HAD	VFD	VD	MW-HAD
	**Aldehydes**							
1	Pentanal	702	705	MS, LRI	92.18 ± 0.23 ^a^	76.12 ± 0.20 ^b^	7.63 ± 0.86 ^c^	6.57 ± 0.68 ^c^
2	Hexanal	816	803	MS, LRI	0.38 ± 0.28 ^c^	/	4.91 ± 1.80 ^b^	15.42 ± 1.99 ^a^
3	Heptanal	919	912	MS, LRI	0.73 ± 0.58 ^ab^	/	4.47 ± 3.05 ^a^	2.71 ± 0.42 ^ab^
4	Methional	925	903	MS, LRI	4.74 ± 0.54 ^a^	2.65 ± 0.12 ^b^	/	/
5	(E)-2-Heptenal	974	960	MS, LRI	2.64 ± 0.20 ^b^	2.10 ± 0.03 ^c^	1.02 ± 0.05 ^d^	6.63 ± 0.06 ^a^
6	Benzaldehyde	979	986	MS, LRI	9.80 ± 0.89 ^a^	9.89 ± 0.84 ^a^	3.37 ± 0.70 ^b^	3.57 ± 0.67 ^b^
7	Octanal	1023	1001	MS, LRI	3.26 ± 1.53 ^a^	/	1.16 ± 0.22 ^b^	3.37 ± 0.04 ^a^
8	Benzeneacetaldehyde	1064	1041	MS, LRI	98.57 ± 1.74 ^b^	70.60 ± 4.58 ^a^	6.76 ± 0.07 ^c^	5.83 ± 0.84 ^c^
9	(E)-2-Octenal	1078	1058	MS, LRI	6.32 ± 1.12 ^b^	38.68 ± 0.27 ^a^	3.22 ± 0.44 ^c^	6.82 ± 0.69 ^b^
10	2-Isopropyl-5-methylhex-2-enal	1122	1106	MS, LRI	/	0.19 ± 0.12 ^a^	/	/
11	Nonanal	1125	1121	MS, LRI	25.95 ± 0.43 ^b^	3.91 ± 0.60 ^c^	4.89 ± 1.04 ^c^	38.35 ± 2.98 ^a^
12	2-Phenylpropenal	1177	1150	MS, LRI	1.56 ± 0.18 ^a^	/	0.21 ± 0.02 ^b^	nd
13	2-Nonenal	1181	1159	MS, LRI	1.61 ± 0.34 ^b^	/	1.49 ± 0.04 ^b^	2.57 ± 0.14 ^a^
14	Decanal	1229	1210	MS, LRI	0.90 ± 0.42 ^b^	1.25 ± 0.19 ^b^	0.78 ± 0.11 ^b^	2.39 ± 0.13 ^a^
15	2,4-Nonadienal	1239	1208	MS, LRI	1.92 ± 0.36 ^a^	/	0.59 ± 0.13 ^b^	0.27 ± 0.20 ^bc^
16	(E)-2-Decenal	1286	1259	MS, LRI	/	/	0.42 ± 0.07 ^b^	1.86 ± 0.95 ^a^
17	2-Phenyl-2-butenal	1292	1273	MS, LRI	1.18 ± 0.14 ^ab^	/	0.18 ± 0.02 ^ab^	2.08 ± 1.58 ^a^
18	Undecanal	1332	1319	MS, LRI	0.20 ± 0.04 ^b^	/	0.10 ± 0.02 ^c^	0.26 ± 0.01 ^a^
19	(E,E)-2,4-Decadienal	1344	1340	MS, LRI	0.71 ± 0.36 ^a^	/	/	/
	Subtotal				252.65	205.39	41.20	98.70
	**Alcohols**							
20	(Z)-2-Penten-1-ol	733	746	MS, LRI	0.81 ± 0.64 ^a^	0.06 ± 0.02 ^b^	/	0.03 ± 0.01 ^b^
21	1-Pentanol	794	769	MS, LRI	/	/	0.57 ± 0.22 ^a^	nd
22	1-Hexanol	890	865	MS, LRI	11.03 ± 1.42 ^a^	13.09 ± 3.76 ^a^	19.34 ± 7.33 ^a^	0.60 ± 0.33 ^b^
23	(E)-2-Hepten-1-ol	990	986	MS, LRI	0.27 ± 0.03 ^b^	0.07 ± 0.02 ^c^	1.70 ± 0.06 ^a^	/
24	3-Octanol	1019	996	MS, LRI	/	56.64 ± 5.38 ^a^	/	/
25	(E)-2-Octen-1-ol	1090	1069	MS, LRI	3.89 ± 0.48 ^a^	/	3.92 ± 0.72 ^a^	4.68 ± 1.68 ^a^
26	1-Octanol	1094	1070	MS, LRI	2.57 ± 0.97 ^b^	17.56 ± 3.01 ^a^	3.23 ± 0.81 ^b^	3.05 ± 1.09 ^b^
27	Phenylethyl alcohol	1137	1110	MS, LRI	0.95 ± 0.43 ^a^	/	0.18 ± 0.06 ^b^	0.21 ± 0.02 ^b^
28	(E)-3-Nonen-1-ol	1171	1143	MS, LRI	0.50 ± 0.17 ^a^	/	0.50 ± 0.02 ^a^	0.14 ± 0.03 ^b^
29	2-Camphanol	1186	1173	MS, LRI	/	/	0.24 ± 0.04 ^a^	/
30	1-Nonanol	1195	1172	MS, LRI	5.64 ± 0.66 ^a^	3.01 ± 0.14 ^b^	2.09 ± 0.15 ^c^	2.52 ± 0.13 ^bc^
31	2-Isopropyl-5-methylcyclohexan-1-ol	1201	1176	MS, LRI	/	/	/	0.20 ± 0.01 ^a^
	Subtotal				25.66	90.43	31.77	11.43
	**Ketones**							
32	2-Hexanone	762	788	MS, LRI	/	/	0.10 ± 0.00 ^b^	3.00 ± 0.49 ^a^
33	3-Heptanone	902	894	MS, LRI	nd	0.43 ± 0.14 ^a^	/	/
34	6-Methyl-2-heptanone	970	956	MS, LRI	0.65 ± 0.16 ^ab^	1.17 ± 0.38 ^a^	0.21 ± 0.02 ^b^	1.40 ± 0.55 ^a^
35	1-Octen-3-one	996	975	MS, LRI	13.94 ± 0.65 ^b^	563.39 ± 58.16 ^a^	/	4.72 ± 0.98 ^b^
36	3-Octanone	1005	988	MS, LRI	/	5716.93 ± 32.59 ^a^	0.25 ± 0.01 ^b^	/
37	3-Octen-2-one	1058	1040	MS, LRI	3.75 ± 0.31 ^b^	6.06 ± 1.54 ^a^	3.07 ± 0.39 ^b^	3.68 ± 0.23 ^b^
38	2-Nonanone	1111	1083	MS, LRI	/	1.42 ± 0.10 ^a^	/	0.64 ± 0.15 ^b^
39	(E,E)-3,5-Octadien-2-one	1114	1098	MS, LRI	/	10.17 ± 1.31 ^a^	/	/
40	Isophorone	1141	1122	MS, LRI	/	/	0.59 ± 0.07 ^a^	/
41	2-Nonen-4-one	1146	1140	MS, LRI	/	/	1.41 ± 0.19 ^a^	1.50 ± 0.37 ^a^
42	(E)-3-Nonen-2-one	1160	1122	MS, LRI	/	/	/	0.07 ± 0.01 ^a^
43	Camphor	1167	1139	MS, LRI	/	0.56 ± 0.38 ^a^	0.47 ± 0.01 ^ab^	0.54 ± 0.20 ^a^
44	1-(4-Methylphenyl)-ethanone	1193	1190	MS, LRI	/	/	1.45 ± 0.01 ^a^	/
45	1-(3-Methylphenyl)-ethanone	1206	1182	MS, LRI	/	/	3.56 ± 0.08 ^a^	/
46	2-Decanone	1213	1190	MS, LRI	/	/	0.32 ± 0.03 ^b^	0.45 ± 0.05 ^a^
47	3-Decen-2-one	1262	1243	MS, LRI	/	/	/	1.13 ± 0.73 ^a^
48	2-Methylene-5-(1-methylethyl)-Cyclohexanone	1272	1238	MS, LRI	/	/	0.34 ± 0.04 ^a^	/
49	Carvenone	1279	1253	MS, LRI	/	/	0.30 ± 0.05 ^a^	/
50	2-Undecanone	1316	1296	MS, LRI	10.41 ± 0.96 ^b^	14.62 ± 2.27 ^a^	4.99 ± 0.61 ^c^	8.95 ± 0.32 ^b^
51	Geranyl acetone	1474	1449	MS, LRI	1.46 ± 0.17 ^ab^	2.29 ± 0.73 ^a^	0.67 ± 0.04 ^b^	1.78 ± 0.09 ^a^
	Subtotal				30.21	6317.04	17.73	27.86
	**Esters**							
52	Methyl butyrate	741	721	MS, LRI	/	/	/	0.92 ± 0.13 ^a^
53	Methyl 2-methylbutyrate	796	770	MS, LRI	/	/	0.07 ± 0.01 ^b^	0.25 ± 0.13 ^a^
54	Heptyl alcohol formate	992	1039	MS, LRI	/	/	10.05 ± 1.98 ^a^	/
55	Ethyl octanoate	1219	1178	MS, LRI	0.65 ± 0.27 ^b^	1.25 ± 0.02 ^a^	/	/
56	Ethenyl hexanoate	1242	1244	MS, LRI	/	/	/	0.70 ± 0.26 ^a^
57	3-Hydroxy-2,2,4-trimethylpentyl isobutyrate	1374	1380	MS, LRI	/	/	/	0.21 ± 0.07 ^a^
58	γ-Nonalactone	1386	1363	MS, LRI	0.04 ± 0.01 ^b^	/	/	0.09 ± 0.02 ^a^
59	(2-Ethyl-3-hydroxyhexyl) 2-Methylpropanoate	1397	1373	MS, LRI	0.13 ± 0.01 ^b^	/	0.14 ± 0.04 ^b^	0.39 ± 0.13 ^a^
60	5-Hydroxy-2-decenoic acid Lactone	1502	1499	MS, LRI	/	/	0.28 ± 0.09 ^a^	0.14 ± 0.06 ^a^
61	2,2,4-Trimethyl-1,3-pentanediol diisobutyrate	1616	1591	MS, LRI	/	/	/	0.24 ± 0.04 ^a^
62	Methyl Pentadecanoate	1863	1820	MS, LRI	1.05 ± 0.22 ^a^	/	/	0.24 ± 0.02 ^b^
63	Dibutyl phthalate	1896	1922	MS, LRI	0.82 ± 0.09 ^a^	/	1.30 ± 0.53 ^a^	/
64	Ethyl Pentadecanoate	1932	1890	MS, LRI	0.70 ± 0.38 ^a^	0.63 ± 0.17 ^a^	0.31 ± 0.20 ^ab^	/
65	Methyl hexadecanoate	1966	1927	MS, LRI	4.87 ± 1.21 ^a^	1.20 ± 0.65 ^b^	1.41 ± 0.24 ^b^	1.46 ± 0.33 ^b^
	Subtotal				8.26	3.08	13.56	4.64
	**Hydrocarbons**							
66	1,3-Octadiene	855	826	MS, LRI	/	385.57 ± 53.09 ^a^	/	/
67	p-Xylene	884	875	MS, LRI	0.54 ± 0.15 ^a^	/	0.22 ± 0.01 ^b^	0.51 ± 0.08 ^a^
68	2-Methyl-2-bornene	1035	1021	MS, LRI	6.24 ± 0.95 ^b^	11.34 ± 0.69 ^a^	0.29 ± 0.08 ^c^	0.89 ± 0.23 ^c^
69	p-Cymene	1042	1023	MS, LRI	/	/	1.26 ± 0.24 ^a^	/
70	4-methyl-1-Undecene	1069	1085	MS, LRI	/	/	0.15 ± 0.05 ^a^	/
71	5-Methyldecane	1075	1057	MS, LRI	1.34 ± 0.18 ^a^	0.91 ± 0.35 ^a^	0.51 ± 0.23 ^a^	1.08 ± 0.56 ^a^
72	2-Methyldecane	1083	1061	MS, LRI	7.57 ± 1.40 ^a^	1.53 ± 0.66 ^bc^	/	2.70 ± 1.07 ^b^
73	3-Methyldecane	1089	1069	MS, LRI	/	65.21 ± 6.44 ^a^	/	/
74	3,5-Dimethyl-Undecane	1221	1207	MS, LRI	5.35 ± 1.64 ^a^	/	/	/
75	1-Methyl-Naphthalene	1319	1325	MS, LRI	/	/	/	0.16 ± 0.12 ^a^
76	3-Methyl-tridecane	1394	1372	MS, LRI	1.15 ± 0.60 ^b^	4.54 ± 2.29 ^a^	0.32 ± 0.02 ^b^	/
77	(Z)-3-Tetradecene	1417	1384	MS, LRI	0.37 ± 0.02 ^a^	/	0.14 ± 0.04 ^b^	/
78	α-Himachalene	1436	1429	MS, LRI	/	/	/	0.76 ± 0.18 ^a^
79	3-Methyl-pentadecane	1597	1570	MS, LRI	1.18 ± 0.48 ^b^	3.40 ± 1.67 ^a^	0.45 ± 0.05 ^b^	0.39 ± 0.07 ^b^
80	5-Phenylundecane	1660	1626	MS, LRI	/	/	0.08 ± 0.02 ^a^	/
81	Undecyl-cyclopentane	1685	1656	MS, LRI	0.66 ± 0.29 ^a^	/	/	/
82	3-Phenyl-undecan	1692	1656	MS, LRI	/	/	0.24 ± 0.00 ^a^	/
83	2-Phenyl-undecan	1731	1692	MS, LRI	/	/	/	0.22 ± 0.08 ^a^
84	6-Phenyldodecane	1755	1719	MS, LRI	/	/	/	0.15 ± 0.02 ^a^
85	Cembrene	1881	1923	MS, LRI	1.90 ± 1.24 ^a^	/	0.72 ± 0.10 ^ab^	0.43 ± 0.10 ^ab^
	Subtotal				26.30	472.50	4.38	7.29
	**Pyrazines**							
86	Tetramethyl-pyrazine	1103	1095	MS, LRI	/	/	/	0.92 ± 0.26 ^a^
87	2,3,5-Trimethyl-6-ethylpyrazine	1174	1163	MS, LRI	/	/	/	0.36 ± 0.05 ^a^
88	2,5-Dimethyl-3-(3-methylbutyl)pyrazine	1335	1310	MS, LRI	0.22 ± 0.09 ^b^	/	0.31 ± 0.02 ^b^	0.55 ± 0.09 ^a^
	Subtotal				0.22	0.00	0.31	1.83
	**Others**							
89	2,4-Di-tert-butylphenol	1542	1512	MS, LRI	0.66 ± 0.42 ^a^	0.75 ± 0.18 ^a^	0.19 ± 0.07 ^a^	0.41 ± 0.16 ^a^
90	4-Tert-Octylphenol	1641	1629	MS, LRI	/	/	3.43 ± 1.98 ^a^	nd
91	Butylated Hydroxytoluene	1531	1483	MS, LRI	0.59 ± 0.40 ^b^	4.20 ± 0.17 ^a^	0.15 ± 0.01 ^b^	0.61 ± 0.27 ^b^
92	Benzothiazole	1249	1224	MS, LRI	/	1.38 ± 0.15 ^a^	/	/
93	1,2,3,4-Tetramethoxybenzene	1560	1533	MS, LRI	2.30 ± 1.25 ^a^	/	/	/
	Subtotal				3.55	6.33	3.77	1.02

^1^ LRI: Linear retention indices calculated by n-alkanes. ^2^ LRI: LRI from the NIST11 database. ^3^ ID: identification methods; MS: mass spectrum fitted to NIST11, WILEY 07 Library; LRI: LRIs of the compounds agreed with database. Different letters (a–d) in the same row meant a significant difference (*p* < 0.05). “/”, not detected.

**Table 4:** Odor activity values (OAVs) of odorants in *Cordyceps militaris* processed by different drying methods.

Compound	Threshold (μg/kg of Water) ^1^	Ref.	OAVs			
			HAD	VFD	VD	MW-HAD
**Aldehydes**						
Pentanal	2.7	[30]	34	28	3	2
Hexanal	4.5	[30]	<1	/	1	3
Heptanal	2.8	[30]	<1	/	2	1
Methional	0.2	[30]	24	13	/	/
Octanal	0.7	[30]	5	/	2	5
Benzeneacetaldehyde	6.3	[30]	16	11	1	1
(E)-2-Octenal	3	[30]	2	13	1	2
Nonanal	1	[30]	26	4	5	38
2-Nonenal	0.1	[30]	16	/	15	26
Decanal	0.3	[30]	3	4	3	8
2,4-Nonadienal	0.05	[30]	38	/	12	5
(E)-2-Decenal	0.3	[30]	/	/	1	6
Undecanal	0.03	[30]	7	/	3	9
(E,E)-2,4-Decadienal	0.027	[30]	26	/	/	/
**Alcohols**						
1-Hexanol	5.6	[30]	2	2	3	<1
3-Octanol	18	[30]	/	3	/	/
Phenylethyl alcohol	0.02	[30]	48	/	9	11
**Ketones**						
1-Octen-3-one	0.016	[50]	871	35212	/	295
3-Octanone	21.4	[30]	/	267	<1	/
2-Undecanone	7	[50]	1	2	<1	1
**Esters**						
Methyl 2-methylbutyrate	0.14	[30]	/	/	<1	2

^1^ Odor threshold in water as reported in the literature reference (Ref.). “/”, not found in the sample.

**Table 5:** Correlation between dried *Cordyceps militaris* samples obtained from GC-IMS.

	Pearson’s Correlation Coefficient											
	HAD-1	HAD-2	HAD-3	VD-1	VD-2	VD-3	VFD-1	VFD-2	VFD-3	MW-HAD-1	MW-HAD-2	MW-HAD-3
**HAD-1**	1	1.000 **	0.999 **	0.586 **	0.584 **	0.557 **	0.045	0.042	0.045	0.417 **	0.438 **	0.400 **
**HAD-2**	1.000 **	1	1.000 **	0.596 **	0.594 **	0.567 **	0.047	0.044	0.047	0.415 **	0.435 **	0.397 **
**HAD-3**	0.999 **	1.000 **	1	0.609 **	0.607 **	0.581 **	0.048	0.045	0.048	0.426 **	0.447 **	0.409 **
**VD-1**	0.586 **	0.596 **	0.609 **	1	1.000 **	0.998 **	0.150	0.148	0.145	0.478 **	0.499 **	0.486 **
**VD-2**	0.584 **	0.594 **	0.607 **	1.000 **	1	0.999 **	0.151	0.149	0.146	0.476 **	0.496 **	0.482 **
**VD-3**	0.557 **	0.567 **	0.581 **	0.998 **	0.999 **	1	0.158	0.156	0.153	0.473 **	0.493 **	0.481 **
**VFD-1**	0.045	0.047	0.048	0.150	0.151	0.158	1	1.000 **	1.000 **	0.636 **	0.549 **	0.536 **
**VFD-2**	0.042	0.044	0.045	0.148	0.149	0.156	1.000 **	1	1.000 **	0.633 **	0.545 **	0.533 **
**VFD-3**	0.045	0.047	0.048	0.145	0.146	0.153	1.000 **	1.000 **	1	0.637 **	0.550 **	0.538 **
**MW-HAD-1**	0.417 **	0.415 **	0.426 **	0.478 **	0.476 **	0.473 **	0.636 **	0.633 **	0.637 **	1	0.992 **	0.976 **
**MW-HAD-2**	0.438 **	0.435 **	0.447 **	0.499 **	0.496 **	0.493 **	0.549 **	0.545 **	0.550 **	0.992 **	1	0.992 **
**MW-HAD-3**	0.400 **	0.397 **	0.409 **	0.486 **	0.482 **	0.481 **	0.536 **	0.533 **	0.538 **	0.976 **	0.992 **	1

The superscript (**) indicates a significant difference at *p* < 0.01.

## Data Availability

The Data presented in this study are contained within the article.

## References

[1] Lao Y., Zhang M., Li Z., Bhandari B. (2022). A novel combination of enzymatic hydrolysis and fermentation: Effects on the flavor and nutritional quality of fermented *Cordyceps militaris* beverage. LWT.

[2] Dong J.Z., Ding J., Yu P.Z., Lei C., Zheng X.J., Wang Y. (2013). Composition and distribution of the main active components in selenium-enriched fruit bodies of *Cordyceps militaris* link. Food Chem..

[3] Zhu Z.Y., Liu F., Gao H., Sun H., Meng M., Zhang Y.M. (2016). Synthesis, characterization and antioxidant activity of selenium polysaccharide from *Cordyceps militaris*. Int. J. Biol. Macromol..

[4] Lin Q., Long L., Wu L., Zhang F., Wu S., Zhang W., Sun X. (2017). Evaluation of different agricultural wastes for the production of fruiting bodies and bioactive compounds by medicinal mushroom *Cordyceps militaris*. J. Sci. Food Agric..

[5] Sun L., Zhang Z., Xin G., Sun B., Bao X., Wei Y., Zhao X., Xu H. (2020). Advances in umami taste and aroma of edible mushrooms. Trends Food Sci. Technol..

[6] Hu S., Feng X., Huang W., Ibrahim S.A., Liu Y. (2020). Effects of drying methods on non-volatile taste components of *Stropharia rugoso-annulata* mushrooms. LWT.

[7] Dong W., Hu R., Chu Z., Zhao J., Tan L. (2017). Effect of different drying techniques on bioactive components, fatty acid composition, and volatile profile of robusta coffee beans. Food Chem..

[8] Ren F., Perussello C.A., Zhang Z., Kerry J.P., Tiwari B.K. (2018). Impact of ultrasound and blanching on functional properties of hot-air dried and freeze dried onions. LWT.

[9] Vega-Galvez A., Poblete J., Rojas-Carmona R., Uribe E., Pasten A., Goni M.G. (2021). Vacuum drying of Chilean papaya (*Vasconcellea pubescens*) fruit pulp: Effect of drying temperature on kinetics and quality parameters. J. Food Sci. Technol..

[10] Jin J., Yurkow E.J., Adler D., Lee T.C. (2018). Improved freeze drying efficiency by ice nucleation proteins with ice morphology modification. Food Res. Int..

[11] Wu F., Tang J., Pei F., Wang S., Chen G., Hu Q., Zhao L. (2015). The influence of four drying methods on nonvolatile taste components of white *Hypsizygus marmoreus*. Eur. Food Res. Technol..

[12] Xu Y., Zhang M., Tu D., Sun J., Zhou L., Mujumdar A.S. (2005). A two-stage convective air and vacuum freeze-drying technique for bamboo shoots. Int. J. Food Sci. Technol..

[13] Li X., Feng T., Zhou F., Zhou S., Liu Y., Li W., Ye R., Yang Y. (2015). Effects of drying methods on the tasty compounds of *Pleurotus eryngii*. Food Chem..

[14] Das I., Arora A. (2018). Alternate microwave and convective hot air application for rapid mushroom drying. J. Food Eng..

[15] Wang Q., Li S., Han X., Ni Y., Zhao D., Hao J. (2019). Quality evaluation and drying kinetics of shitake mushrooms dried by hot air, infrared and intermittent microwave-assisted drying methods. LWT.

[16] Tian Y., Zhao Y., Huang J., Zeng H., Zheng B. (2016). Effects of different drying methods on the product quality and volatile compounds of whole shiitake mushrooms. Food Chem..

[17] Louw S. (2021). Recent trends in the chromatographic analysis of volatile flavor and fragrance compounds: Annual review 2020. Anal. Sci. Adv..

[18] Wu X.F., Zhang M., Bhandari B. (2019). A novel infrared freeze drying (IRFD) technology to lower the energy consumption and keep the quality of *Cordyceps militaris*. Innov. Food Sci. Emerg. Technol..

[19] Wu X.F., Zhang M., Li Z. (2019). Influence of infrared drying on the drying kinetics, bioactive compounds and flavor of *Cordyceps militaris*. LWT.

[20] Wu X.F., Zhang M., Bhandari B., Li Z. (2018). Effects of microwave-assisted pulse-spouted bed freeze-drying (MPSFD) on volatile compounds and structural aspects of *Cordyceps militaris*. J. Sci. Food Agric..

[21] Vautz W., Franzke J., Zampolli S., Elmi I., Liedtke S. (2018). On the potential of ion mobility spectrometry coupled to GC pre-separation-A tutorial. Anal. Chim. Acta.

[22] Wang S., Chen H., Sun B. (2020). Recent progress in food flavor analysis using gas chromatography-ion mobility spectrometry (GC-IMS). Food Chem..

[23] Hou H., Liu C., Lu X., Fang D., Hu Q., Zhang Y., Zhao L. (2021). Characterization of flavor frame in shiitake mushrooms (*Lentinula edodes*) detected by HS-GC-IMS coupled with electronic tongue and sensory analysis: Influence of drying techniques. LWT.

[24] Chen D., Qin L., Geng Y., Kong Q., Wang S., Lin S. (2021). The Aroma Fingerprints and Discrimination Analysis of Shiitake Mushrooms from Three Different Drying Conditions by GC-IMS, GC-MS and DSA. Foods.

[25] Zeng X., Liu J., Dong H., Bai W., Yu L., Li X. (2020). Variations of volatile flavour compounds in *Cordyceps militaris* chicken soup after enzymolysis pretreatment by SPME combined with GC-MS, GC × GC-TOF MS and GC-IMS. Int. J. Food Sci. Technol..

[26] Lan X., Liu P., Xia S., Jia C., Mukunzi D., Zhang X., Xia W., Tian H., Xiao Z. (2010). Temperature effect on the non-volatile compounds of Maillard reaction products derived from xylose-soybean peptide system: Further insights into thermal degradation and cross-linking. Food Chem..

[27] Pei F., Shi Y., Gao X., Wu F., Mariga A.M., Yang W., Zhao L., An X., Xin Z., Yang F. (2014). Changes in non-volatile taste components of button mushroom (*Agaricus bisporus*) during different stages of freeze drying and freeze drying combined with microwave vacuum drying. Food Chem..

[28] Qi J., Xu Y., Zhang W., Xie X., Xiong G., Xu X. (2021). Short-term frozen storage of raw chicken meat improves its flavor traits upon stewing. LWT.

[29] Wang P., Liu J., Chen D.W. (2021). Analysis of aroma-active compounds in bighead carp head soup and their influence on umami of a model soup. Microchem. J..

[30] Van Gemert L.J. (2011). Odour Thresholds: Compilations of Odour Threshold Values in Air, Water and Other Media.

[31] Yu X., Chen X., Li Y., Li L. (2022). Effect of Drying Methods on Volatile Compounds of *Citrus reticulata* Ponkan and Chachi Peels as Characterized by GC-MS and GC-IMS. Foods.

[32] Fernandes Â., Barros L., Barreira J.C.M., Antonio A.L., Oliveira M.B.P.P., Martins A., Ferreira I.C.F.R. (2013). Effects of different processing technologies on chemical and antioxidant parameters of *Macrolepiota procera* wild mushroom. LWT.

[33] Fang D., Yang W., Benard M.K., Zhao L., An X., Hu Q. (2017). Comparison of flavour qualities of mushrooms (*Flammulina velutipes*) packed with different packaging materials. Food Chem..

[34] Li W., Gu Z., Yang Y., Zhou S., Liu Y., Zhang J. (2014). Non-volatile taste components of several cultivated mushrooms. Food Chem..

[35] Liu Y., Huang F., Yang H., Ibrahim S.A., Wang Y.F., Huang W. (2014). Effects of preservation methods on amino acids and 5’-nucleotides of *Agaricus bisporus* mushrooms. Food Chem..

[36] Leksrisompong P., Gerard P., Lopetcharat K., Drake M. (2012). Bitter taste inhibiting agents for whey protein hydrolysate and whey protein hydrolysate beverages. J. Food Sci..

[37] Chen W., Li W., Yang Y., Yu H., Zhou S., Feng J., Li X., Liu Y. (2015). Analysis and evaluation of tasty components in the pileus and stipe of *Lentinula edodes* at different growth stages. J. Agric. Food Chem..

[38] Dong M., Qin L., Xue J., Du M., Lin S.Y., Xu X.B., Zhu B.W. (2018). Simultaneous quantification of free amino acids and 5’-nucleotides in shiitake mushrooms by stable isotope labeling-LC-MS/MS analysis. Food Chem..

[39] Zhang L., Dong X., Feng X., Ibrahim S.A., Huang W., Liu Y. (2021). Effects of Drying Process on the Volatile and Non-Volatile Flavor Compounds of *Lentinula edodes*. Foods.

[40] Qi J., Liu D.Y., Zhou G.H., Xu X.L. (2017). Characteristic Flavor of Traditional Soup Made by Stewing Chinese Yellow-Feather Chickens. J. Food Sci..

[41] Aisala H., Sola J., Hopia A., Linderborg K.M., Sandell M. (2019). Odor-contributing volatile compounds of wild edible Nordic mushrooms analyzed with HS-SPME-GC-MS and HS-SPME-GC-O/FID. Food Chem..

[42] Li M., Yang R., Zhang H., Wang S., Chen D., Lin S. (2019). Development of a flavor fingerprint by HS-GC-IMS with PCA for volatile compounds of Tricholoma matsutake Singer. Food Chem..

[43] Cho I.H., Kim S.Y., Choi H.K., Kim Y.S. (2006). Characterization of aroma-active compounds in raw and cooked pine-mushrooms (*Tricholoma matsutake* Sing.). J. Agric. Food Chem..

[44] Wang W., Feng X., Zhang D., Li B., Sun B., Tian H., Liu Y. (2018). Analysis of volatile compounds in Chinese dry-cured hams by comprehensive two-dimensional gas chromatography with high-resolution time-of-flight mass spectrometry. Meat Sci..

[45] Li Q., Zhang L., Li W., Li X., Huang W., Yang H., Zheng L. (2016). Chemical compositions and volatile compounds of Tricholoma matsutake from different geographical areas at different stages of maturity. Food Sci. Biotechnol..

[46] Cullere L., Ferreira V., Venturini M.E., Marco P., Blanco D. (2013). Potential aromatic compounds as markers to differentiate between Tuber melanosporum and Tuber indicum truffles. Food Chem..

[47] Liu L., Chen Y., Luo Q., Xu N., Zhou M., Gao B., Wang C., Shi Y. (2018). Fermenting liquid vinegar with higher taste, flavor and healthy value by using discarded *Cordyceps militaris* solid culture medium. LWT.

[48] Guo Y., Chen D., Dong Y., Ju H., Wu C., Lin S. (2018). Characteristic volatiles fingerprints and changes of volatile compounds in fresh and dried Tricholoma matsutake Singer by HS-GC-IMS and HS-SPME-GC-MS. J. Chromatogr. B.

[49] Zhuang K., Wu N., Wang X., Wu X., Wang S., Long X., Wei X. (2016). Effects of 3 Feeding Modes on the Volatile and Nonvolatile Compounds in the Edible Tissues of Female Chinese Mitten Crab (*Eriocheir sinensis*). J. Food Sci..

[50] Zhang H., Pu D., Sun B., Ren F., Zhang Y., Chen H. (2018). Characterization and comparison of key aroma compounds in raw and dry porcini mushroom (*Boletus edulis*) by aroma extract dilution analysis, quantitation and aroma recombination experiments. Food Chem..

[51] Gerhardt N., Birkenmeier M., Sanders D., Rohn S., Weller P. (2017). Resolution-optimized headspace gas chromatography-ion mobility spectrometry (HS-GC-IMS) for non-targeted olive oil profiling. Anal. Bioanal. Chem..

[52] Yang Y., Wang B., Fu Y., Shi Y.G., Chen F.L., Guan H.N., Liu L.L., Zhang C.Y., Zhu P.Y., Liu Y. (2021). HS-GC-IMS with PCA to analyze volatile flavor compounds across different production stages of fermented soybean whey tofu. Food Chem..

